# Identification of potential prognostic biomarkers of thymoma with myasthenia gravis based on serum proteomics

**DOI:** 10.3389/fimmu.2025.1580219

**Published:** 2025-06-17

**Authors:** Xiaoting Lin, Peng Liu, Guoyan Qi

**Affiliations:** ^1^ Department of Oncology, Hebei Medical University, Shijiazhuang, Hebei, China; ^2^ Treatment Center of Myasthenia Gravis, People’s Hospital of Shijiazhuang, Shijiazhuang, Hebei, China; ^3^ Hebei Provincial Key Laboratory of Myasthenia Gravis, Shijiazhuang, Hebei, China; ^4^ Hebei Provincial Clinical Research Center for Myasthenia Gravis, Shijiazhuang, Hebei, China

**Keywords:** thymoma, myasthenia gravis, proteomics, prognostic, biomarkers

## Abstract

**Background:**

Thymoma is often associated with myasthenia gravis (MG), and the resection of thymoma improves myasthenic symptoms in patients with thymoma and MG (TMG), but some patients still have no relief. Through proteomic analysis, we examined preoperative serum samples from patients with TMG to identify key prognostic proteins that could serve as a foundation for clinically predicting postoperative efficacy and guiding treatment selection.

**Method:**

According to the Clinical Research Guidelines of the Myasthenia Gravis Foundation of America (MGFA) for Post-Intervention Status (PIS), 20 patients with TMG were divided into an effective group (T1) [the PIS was minimal manifestation status (MMS) and above] and an ineffective group (T2) (the PIS did not reach MMS and above), with 10 cases each, and a healthy control group (C) with nine cases. Blood samples from the three groups were collected through data-independent acquisition (DIA) proteomic analysis performed by mass spectrometry to identify differentially expressed proteins and search for key proteins associated with myasthenia prognosis. Finally, the target proteins were validated through the utilization of enzyme-linked immunosorbent assay (ELISA).

**Results:**

A total of 514 proteins were identified in this research. Between the T1 and T2 groups, there were 20 proteins that exhibited differential expression, with 10 showing upregulation and 10 displaying downregulation. The Kyoto Encyclopedia of Genes and Genomes (KEGG) functional annotation indicated that these proteins were mainly involved in signaling pathways such as complement and coagulation cascade, prion disease, systemic lupus erythematosus, neutrophil extracellular trap formation, and transcription dysregulation in cancer. Three proteins were discovered to have a significant correlation with the prognosis of TMG: L-selectin (SELL) was downregulated, and human leukocyte antigen (HLA) class I histocompatibility antigen (HLA-A) and complement 5 (C5) were upregulated. ELISA results confirmed the proteomic results.

**Conclusion:**

HLA-A, C5, and SELL may be potential prognostic biomarkers of TMG. This study may provide a more accurate prognostic risk assessment of TMG patients to help clinicians better individualize the initial treatment regimen for patients with different risk stratification, plan a more reasonable frequency of follow-up visits, and make more precise maintenance treatment decisions, thereby improving the overall prognosis of TMG patients.

## 1 Introduction

Thymoma is a rare type of tumor that develops in the anterior mediastinum and has a unique association with autoimmune diseases. Approximately one-third of thymoma patients have an autoimmune disease, and the most common concomitant disease is myasthenia gravis (MG) (1). MG is an autoimmune disease that is characterized by acquired dysfunction in neuromuscular junction transmission due to the presence of autoantibodies (2). Studies have indicated that thymoma with MG (TMG) exhibits a more unfavorable prognosis (3).

According to the most recent guidelines, surgical resection is mandatory in TMG patients, with few exceptions, such as inoperable tumors, older age, and/or multimorbidity (4). The clinical symptoms of MG were improved or cured in some patients after thymoma resection (5), but there were also some patients with poor postoperative symptom relief or postoperative MG symptom relapse. The proportion of MG symptom relief after thymectomy has been reported to vary widely, from 9.6% to 57.5% (6). The pathogenesis of TMG is complex, and prognostic biomarkers have not yet been identified. The fluctuating disease dynamics of molecular synthesis and degradation, resulting in high variability of the biological responses, hinder the discovery of reliable biomarkers (7). Although the measurement of anti-acetylcholine receptor antibodies (AChRab) is valuable in the identification and serological categorization of patients with MG, according to literature, AChRab titers cannot indeed reflect the prognosis of patients; however, IgG1 subtype titers are to some extent correlated with the severity of myasthenic symptoms (8–12). Early prediction is important for the individual treatment of patients; however, at present, there are no biomarkers that can reinforce therapeutic decision-making in patient management. Ideal biomarkers should be easy to measure, can objectively and accurately obtain results, have high sensitivity and specificity, and can provide information about the future development, outcome, or survival of the disease, which can help doctors predict the course of the disease, the patient’s response to treatment, the possibility of recurrence, the overall survival time, and other important prognostic information. Therefore, we are looking for biological markers that reflect the prognosis of TMG, can predict the clinical changes of the disease in patients, and support clinical treatment decisions.

Proteins are the actual executors of cellular life activities, and therefore, they can more accurately define the disease state than genomics alone, even though the proteome is highly dynamic (13). Currently, the majority of clinical research and practice rely on antibody-based methods, such as immunohistochemistry and enzyme-linked immunosorbent assay (ELISA), for the detection and quantification of proteins (14). Although these methods are generally reliable and have been widely used, they are restricted to known individual proteins and therefore provide a limited amount of information (15). Proteomics is the study of proteins encoded by the genome of an organism and expressed at a given state (13, 16). Proteomics plays an important role in disease categorization, the determination of treatment targets, the forecasting of treatment outcomes, and prognostic assessment (15). In discovery-oriented proteomics, there are two widely adopted data acquisition strategies mdash;data-dependent acquisition (DDA) and data-independent acquisition (DIA)—that mainly differ in mass spectrometry scanning modes. DIA mass spectrometry is a powerful technology for high-throughput, accurate, and reproducible quantitative proteomics (17). In this study, DIA mass spectrometry was used to analyze preoperative sera from TMG patients, identifying key proteins associated with prognosis. Hence, this study may provide a more accurate prognostic risk assessment of TMG patients to help clinicians better individualize the initial treatment regimen for patients with different risk stratification, plan a more reasonable frequency of follow-up visits, and make more precise maintenance treatment decisions, thereby improving the overall prognosis of TMG patients.

## 2 Materials and methods

### 2.1 Inclusion criteria for patients

Twenty patients with TMG were diagnosed in Shijiazhuang People’s Hospital affiliated with Hebei Medical University from July 2017 to December 2019, underwent thoracoscopic thymoma resection, and were followed up for 5 years. The diagnostic criteria of MG were typical clinical characteristics of MG, positive neostigmine test, and serum anti-AChR antibody detection positivity (18). The diagnosis of thymoma was conducted using postoperative pathology reports. Patients were excluded if they had one of the following conditions: 1) received radiotherapy, chemotherapy, targeted drugs, and immunosuppression treatments; 2) complicated with other tumors or autoimmune diseases; or 3) thymoma metastasis or recurrence occurred after surgery. According to the Clinical Research Guidelines of the Myasthenia Gravis Foundation of America (MGFA) for Post-Intervention Status (PIS), they were divided into an effective group (T1) [the PIS was minimal manifestation status (MMS) and above] and an ineffective group (T2) (the PIS did not reach MMS and above) with 10 cases each. The healthy control group (C) had nine cases. The inclusion criteria for the healthy control group were as follows: over 18 years old and had no hypertension, diabetes, heart disease, autoimmune diseases, or tumors. The exclusion criteria were as follows: under 18 years old and pregnant. An additional 30 patients with TMG were enrolled for the validation of key proteins; they were divided into the T1 and T2 groups according to the above grouping criteria, with 15 patients each. This study was approved by the hospital’s ethics committee. All of the participants gave their informed consent and were approved by the ethics committee.

### 2.2 Serum preparation of mass spectrometry samples

The blood samples were collected from 20 patients with TMG (1 day before surgery) and nine healthy controls in the morning. Additionally, from the ELISA validation group, 30 patients’ preoperative blood samples were collected. Blood samples were centrifuged at 4,000 r/min for 5 minutes at room temperature to collect the serum supernatant. The serum samples were diluted with binding buffer (Millipore Corp., Burlington, MA, USA), and samples after albumin/IgG removal were freeze-dried under vacuum, followed by the addition of 250 μL sodium dodecyl sulfate (SDS) for re-dissolution. The supernatant was obtained as the sample’s total protein solution through centrifugation at 12,000 ×*g* for 10 minutes at room temperature. For subsequent experiments, 50 μg of protein was utilized. dithiothreitol (DTT) was introduced into the solution and then incubated at 55°C for 30 minutes. An appropriate amount of iodoacetamide was added to the mixture, with the final concentration of 9 mM, thoroughly mixed, and kept in a dark area for 15 minutes. The protein was extracted utilizing acetone precipitation and then stored at −20°C overnight. The sample was centrifuged at 8,000 ×*g* for 10 minutes at 4°C to obtain the precipitate. The precipitate was dissolved using 100 μL of triethylammonium bicarbonate (TEAB), and Trypsin-TPCK with a concentration of 1 mg/mL was introduced for digestion at 37°C overnight. The enzymolysis reaction was terminated by the addition of phosphoric acid to adjust the pH value to approximately 3, and the mixture was subsequently subjected to a desalting process.

### 2.3 Data-independent acquisition LC-MS/MS-based proteomic detection

The separation of components was conducted using an Agilent 1100 in a mobile phase with pH 10. The determination was performed on a narrow-diameter Agilent Zorbax Extend-C18 column (5 μm, 150 mm × 2.1 mm). Mobile phase A was composed of a mixture of 2% acetonitrile and water, while mobile phase B was prepared by combining 90% acetonitrile with water. Each component was separated by a C18 column on the EASY-nLC™ 1200 system. The Q-Exactive HF-X mass spectrometer (Thermo Fisher, Waltham, MA, USA) was utilized in the study. The flow rate was adjusted to 300 NL/min, and the chromatographic gradient lasted for 90 minutes.

The MS output was compared with the theoretical spectrum generated by the fasta library, enabling the conversion of the MS signal into valuable peptide and protein sequence data. For the integration of sequence data, peptide elution time, and fragment ion details, a spectrum library was established to facilitate subsequent DIA analysis. The Spectronaut Pulsar was utilized to align the processing of DIA raw data.

To perform subsequent calculations, proteins with an expression level exceeding 50% in each group were merged directly. Conversely, proteins exhibiting less than 50% expression were averaged within their respective groups. Next, the credible proteins were acquired through peak area standardization and log_2_ logarithmic transformation. The screening criteria for differentially expressed proteins (DEPs) were *p* < 0.05, and the fold change (FC) was <0.83 or >1.20.

### 2.4 Bioinformatics analysis

To gain insights into the biological significance of proteins that have been identified, functional annotation and enrichment analysis were conducted using the Gene Ontology (GO) and Kyoto Encyclopedia of Genes and Genomes (KEGG) database, the analysis of protein–protein interaction (PPI) was conducted using the String database (v11.5, https://cn.string-db.org/), and the network visualization was performed using the Cytoscape software (v3.7.2).

### 2.5 HLA-A, SELL, and C5 measurement

HLA-A, SELL, and C5 in the serum were determined using a human HLA-A ELISA kit (ZC-33970, zcibio, Shanghai, China.), a human SELL ELISA kit (ZC-31978, zcibio, Shanghai, China.), and a human C5 ELISA kit (ZC-32561, zcibio, Shanghai, China.). ELISA was performed according to the manufacturer’s instructions.

### 2.6 Statistical analyses

DEPs from the T1 and T2 groups were screened, and the t-test was used to calculate the significance of DEPs. The statistical analysis of ELISA results was performed utilizing the GraphPad Prism Software version 9.0, with a significance level set at *p* < 0.05.

## 3 Results

### 3.1 Patients’ basic information

Our study included a total of 29 patients, including the effective group (T1, N = 10), the ineffective group (T2, N = 10), and the healthy control group (C, N = 9), all of whom had no underlying diseases such as hypertension, diabetes, or other autoimmune disorders. There were no statistically significant differences observed in age, sex, MGFA classification, and pathological type in all groups. The data of patients and healthy participants are shown in **Table 1**.

**Table 1:** Baseline characteristics of patients.

Baseline characteristics	Classification	Control (C) N=9	the effective group (T1, N=10)	the ineffective group (T2, N=10)	*P* value
Gender (n %)	Male	5(55.56)	5(50)	6(60)	1.000
	Female	4(44.44)	5(50)	4(40)	
Age	years, mean ± SD	38±1.6	48±10.27	46.8±8.19	0.776
AchRab	nmol/L, mean ± SD	<0.01	8.49±4.69	9.99±3.93	0.448
MGFA classification	I		1	1	0.807
	IIa, IIb		5	3	
	IIIa, IIIb		3	4	
	IVa, IVb		1	2	
WHO pathological type	AB		2	1	0.548
	B1		1	1	
	B2		5	3	
	B3		2	5	

AchRab, Anti-acetylcholine receptor antibodies; MGFA, Myasthenia Gravis Foundation of America; SD, standard deviation; WHO, World Health Organization.

### 3.2 Proteomic analysis

**Figure 1:**
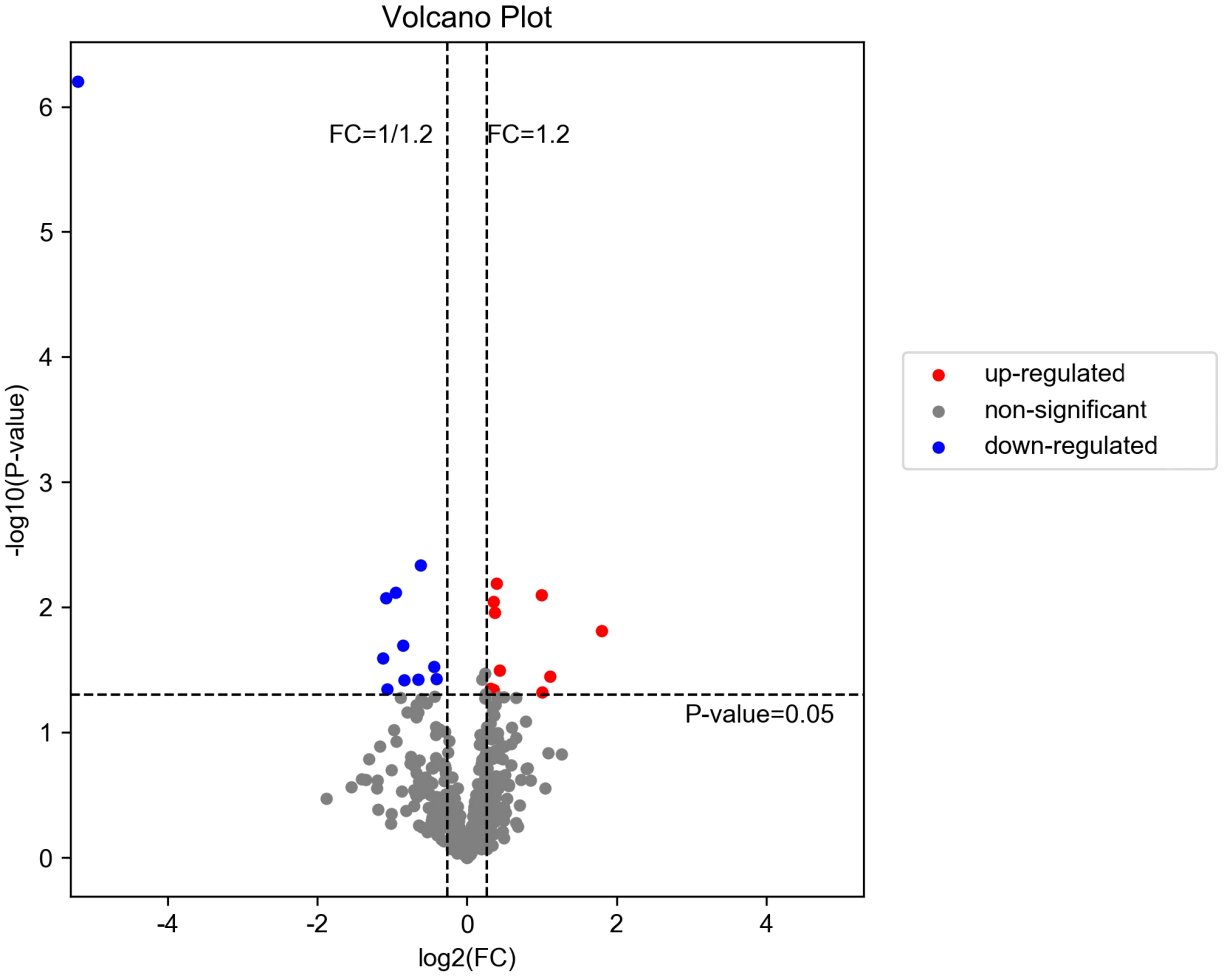
Volcano diagram of DEPs in T1 vs. T2. The downregulated differentially expressed proteins (DEPs) are represented by the blue dot in the image, while the upregulated ones are indicated by the red dot. The non-significant differentially expressed proteins can be identified through the gray dot.

**Figure 2:**
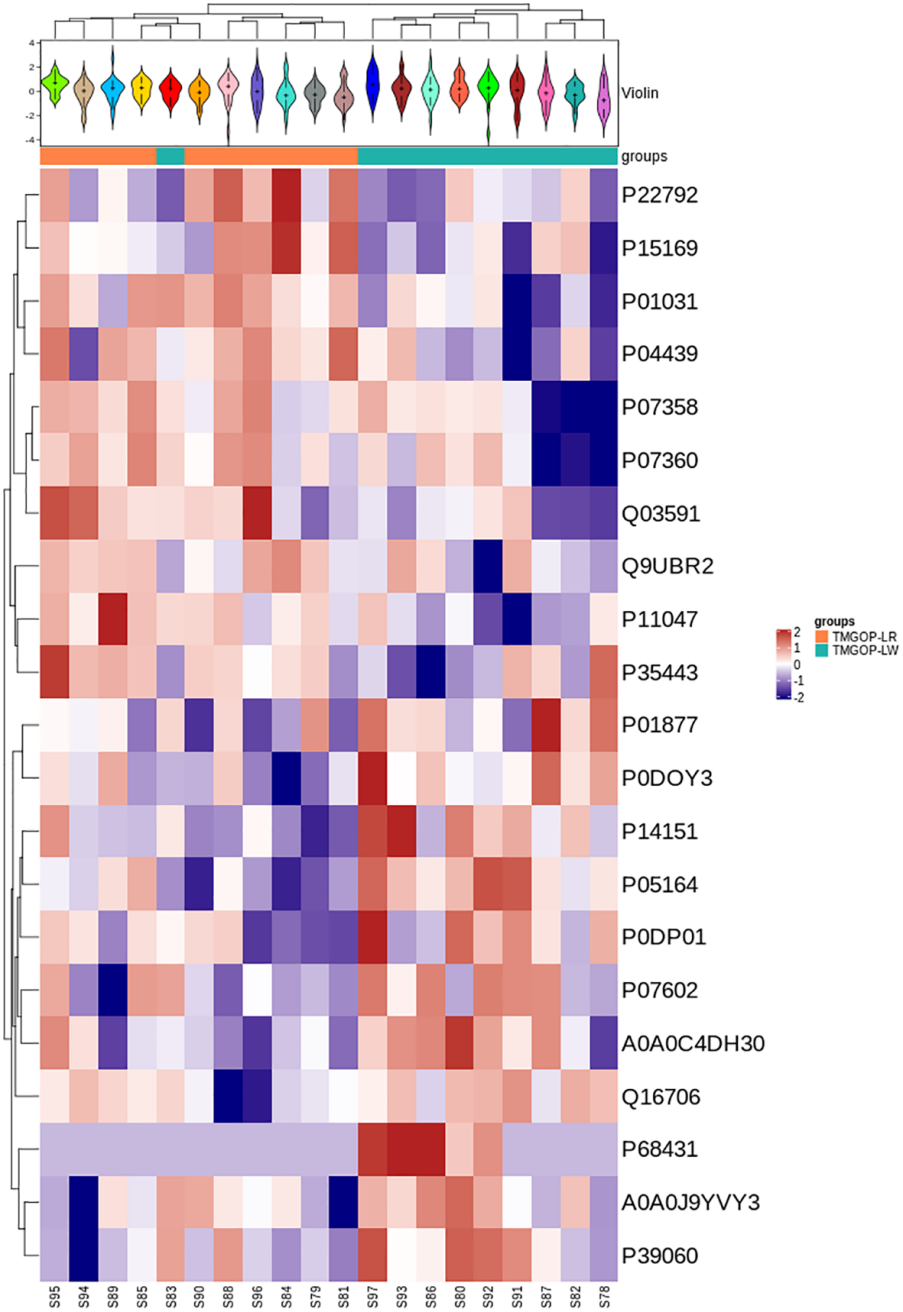
Expression pattern clustering heat map. At the top is the violin plot, combining a box plot and density plot. Flatter sections indicate higher data concentration, showing the probability distribution of protein expression values. Different colors represent distinct samples. The “+” symbol marks the median value, while the y-axis shows protein expression levels. At the bottom is a heat map with column annotations, where colored blocks indicate sample groups. The heat map clusters proteins by expression level, using red for high expression and blue for low expression.

A total of 514 serum proteins were found in the T1 and T2 groups. FC was used to evaluate the multiples of changes in protein expression levels between the two groups: FC = The average value of T1 group protein/The average value of T2 group protein. The *p*-value calculated using the t-test shows the significance of the differences between the two groups. The screening conditions for DEPs were as follows: FC <0.83 or >1.20 and *p* < 0.05. Twenty DEPs were obtained, with 10 showing upregulation (FC > 1.2 and *p* < 0.05) and the remaining 10 exhibiting downregulation (FC < 0.83 and *p* < 0.05). Between the two groups, the corresponding gene names, protein descriptions, *p*-values, and FC values of all differential proteins are shown in **Table 2**. The volcano plots and clustering heat maps of differential protein expression are shown in **Figures 1** and **2**, respectively.

**Table 2:** Differential protein information.

Protein descriptions	Protein names	*P*-Value	FC	State
Immunoglobulin heavy variable 3-16	IGHV3-16	0.025635727	0.458549439	Down
Collagen alpha-1 (XVIII) chain	COL18A1	0.00844173	0.47050426	Down
Alpha-mannosidase 2	MAN2A1	0.045297597	0.475803524	Down
Myeloperoxidase	MPO	0.007639785	0.517608452	Down
Prosaposin	PSAP	0.020137672	0.552958028	Down
Immunoglobulin heavy variable 1-8	IGHV1-8	0.038145163	0.559562822	Down
Immunoglobulin heavy variable 7-4-1	IGHV7-4-1	0.037877455	0.635266069	Down
L-selectin	SELL	0.004594287	0.651187534	Down
Immunoglobulin lambda constant 3	IGLC3	0.029716764	0.734099602	Down
Immunoglobulin heavy constant alpha 2	IGHA2	0.037163256	0.752778327	Down
Complement component 8 beta chain	C8B	0.044826363	1.244653474	Up
Carboxypeptidase N catalytic chain	CPN1	0.00899506	1.275996288	Up
Complement component 8 gamma chain	C8G	0.045537962	1.277148538	Up
Complement 5	C5	0.011088652	1.293647753	Up
Carboxypeptidase N subunit 2	CPN2	0.006449199	1.313220231	Up
Complement factor H-related protein 1	CFHR1	0.031869187	1.353444991	Up
HLA class I histocompatibility antigen, A alpha chain	HLA-A	0.00799727	1.994834424	Up
Cathepsin Z	CTSZ	0.047660306	2.006798529	Up
Thrombospondin-4	THBS4	0.035881945	2.158005624	Up
Laminin subunit gamma-1	LAMC1	0.015450881	3.491517083	Up

FC, fold change.

### 3.3 GO and KEGG functional annotation and enrichment analysis

**Figure 3:**
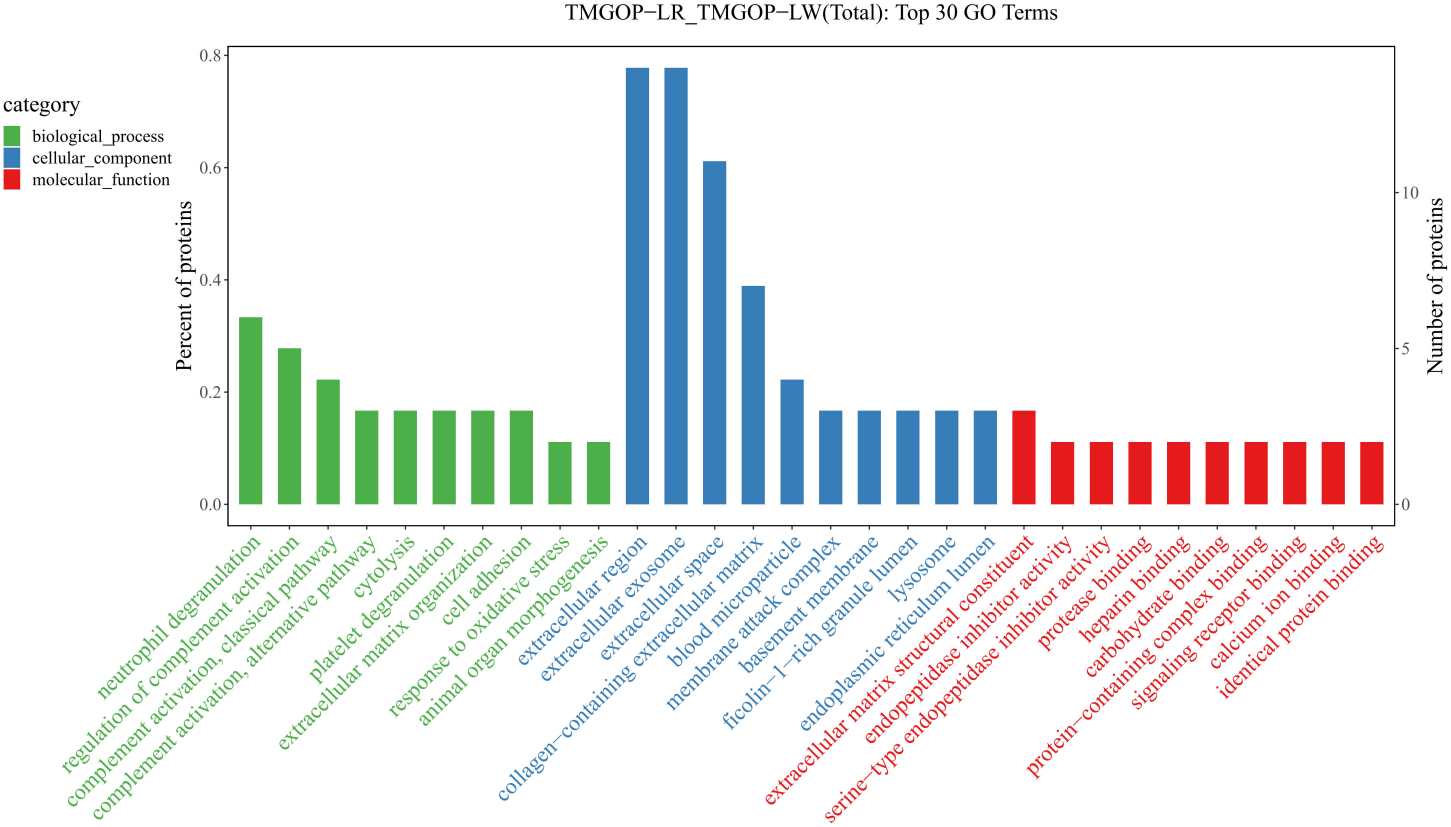
Results of GO enrichment/GO enrichment and classification bar chart. The x-coordinate represents the GO entry’s name, whereas the y-coordinate indicates the count and proportion of proteins within that particular entry. GO, Gene Ontology.

**Figure 4:**
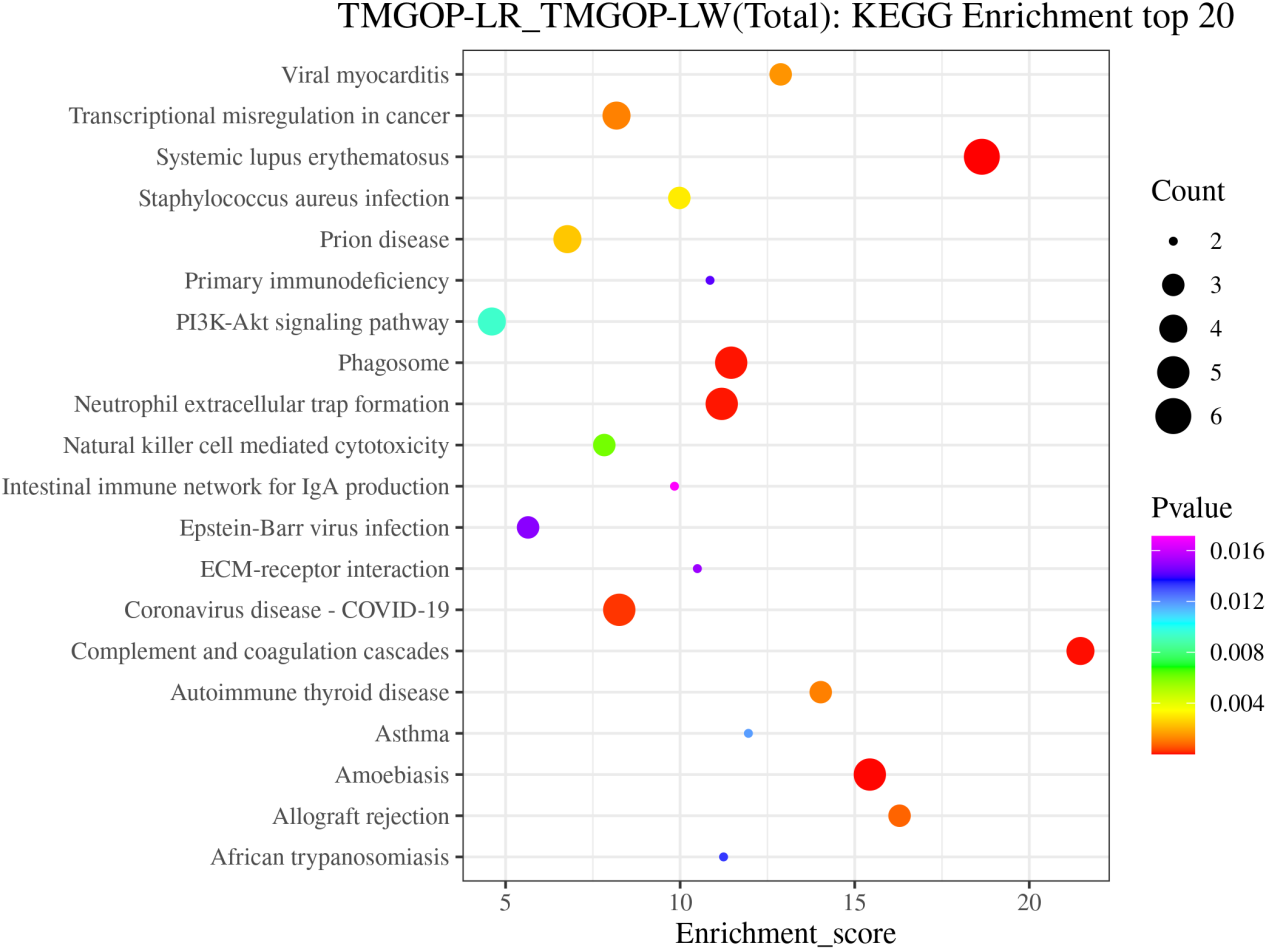
KEGG annotation and enrichment analysis. The enrichment score is depicted on the x-coordinate, while the pathway information of the top 20 proteins is represented by the y-coordinate. The size of the bubbles corresponds directly to the amount of proteins that show differential expression, and their color gradient ranges from red to green, blue, and purple. More significant enrichments are indicated by smaller *p*-values. KEGG, Kyoto Encyclopedia of Genes and Genomes.

The possible roles of DEPs were further predicted through the implementation of GO/KEGG enrichment function analysis, and the identified differential proteins were categorized and annotated according to three distinct aspects: biological processes, cellular components, and molecular functions. GO items with a corresponding differential protein number greater than 1 in the three categories were screened, and the bar chart is shown in **Figure 3**. In the biological process, DEPs were primarily involved in the regulation of complement activation and the complement activation, classical pathway. The primary cellular components exhibiting enrichment of DEPs encompassed the extracellular region, extracellular exosomes, and extracellular space. The DEPs exhibited enrichment in molecular functions related to the binding of antigens and receptors for immunoglobulins. The major pathways involved in differential proteins are systemic lupus erythematosus and complement and coagulation cascade (**Figure 4**).

### 3.4 PPI network construction

**Figure 5:**
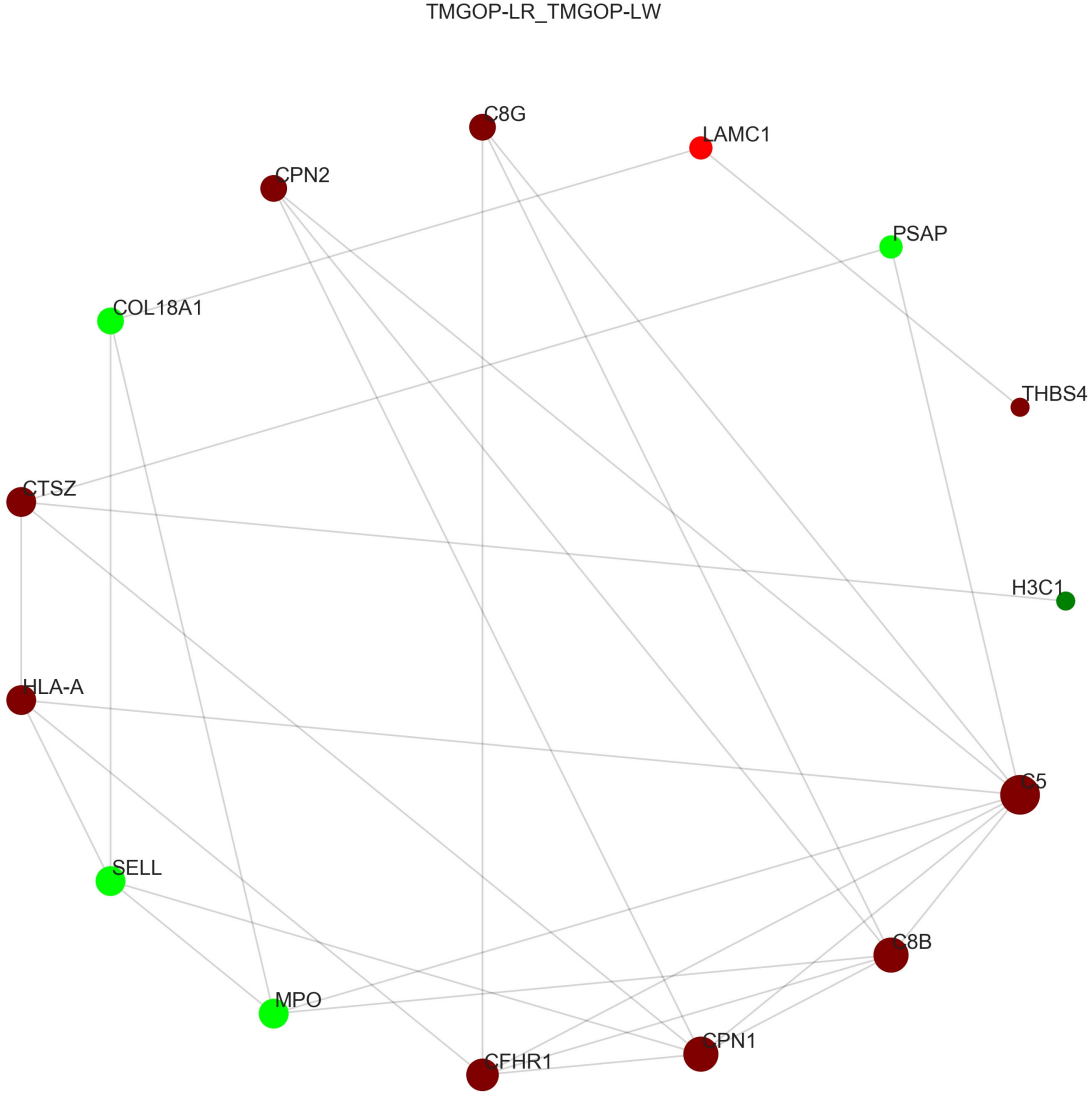
Protein interaction network diagram. The circle represents differential proteins, with red indicating upregulated proteins and green indicating downregulated proteins. The size of each circle corresponds to the degree of connectivity, with larger circles indicating higher levels of connectivity.

The interaction of DEPs was evaluated using the STRING database, and the protein–protein interaction network diagram was created through the utilization of the Cytoscape software; the results are shown in **Figure 5**. In the picture, the circle represents differential proteins, with red indicating upregulated proteins and green indicating downregulated proteins. The size of each circle corresponds to the degree of connectivity, with larger circles indicating higher levels of connectivity. Proteins with high connectivity may be the key points affecting the metabolism or signal transduction pathways of the whole system, and they are candidate proteins for subsequent focused research. The results indicated that C5 is the protein with the highest connectivity and has significant interactions with other complement components such as C8B, C8G, and CHFR-1. C5 plays an important role in the pathogenesis of myasthenia gravis; this finding provides important information for our research direction.

### 3.5 ELISA-based validation of candidate biomarkers

**Figure 6:**
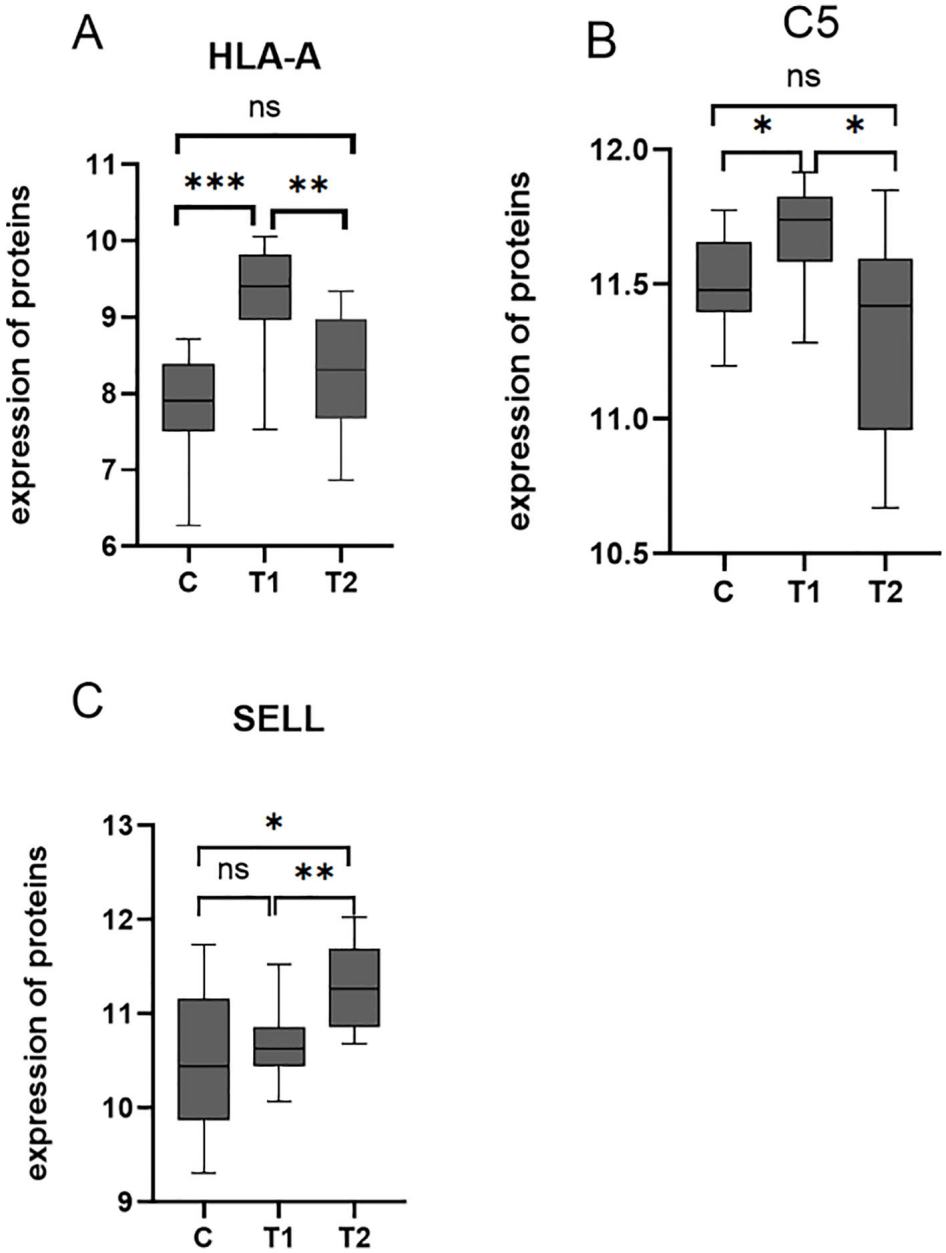
**(A–C)** Box plots of the expression of HLA-A, C5, and SELL in C, T1, and T2 groups (*p*-values were calculated using t-test; *p < 0.05, **p < 0.01, and ***p < 0.001) ns, not significant.

**Figure 7:**
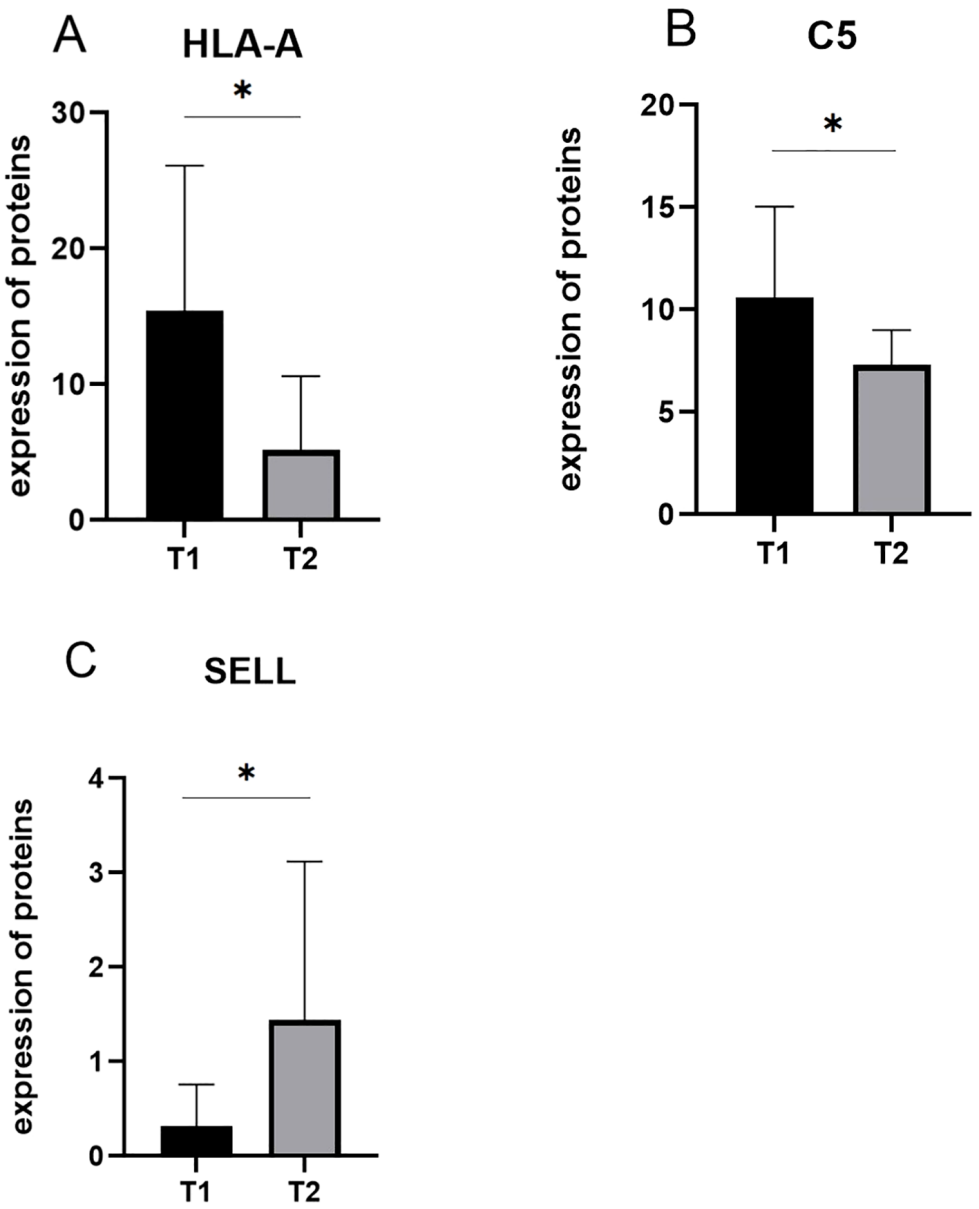
**(A–C)** The expression of candidate proteins was validated by ELISA. C5 and HLA-A were upregulated in the T1 group, while SELL was downregulated in the T1 group (*p*-values were calculated using t-test; *p < 0.05).

**Figure 8:**
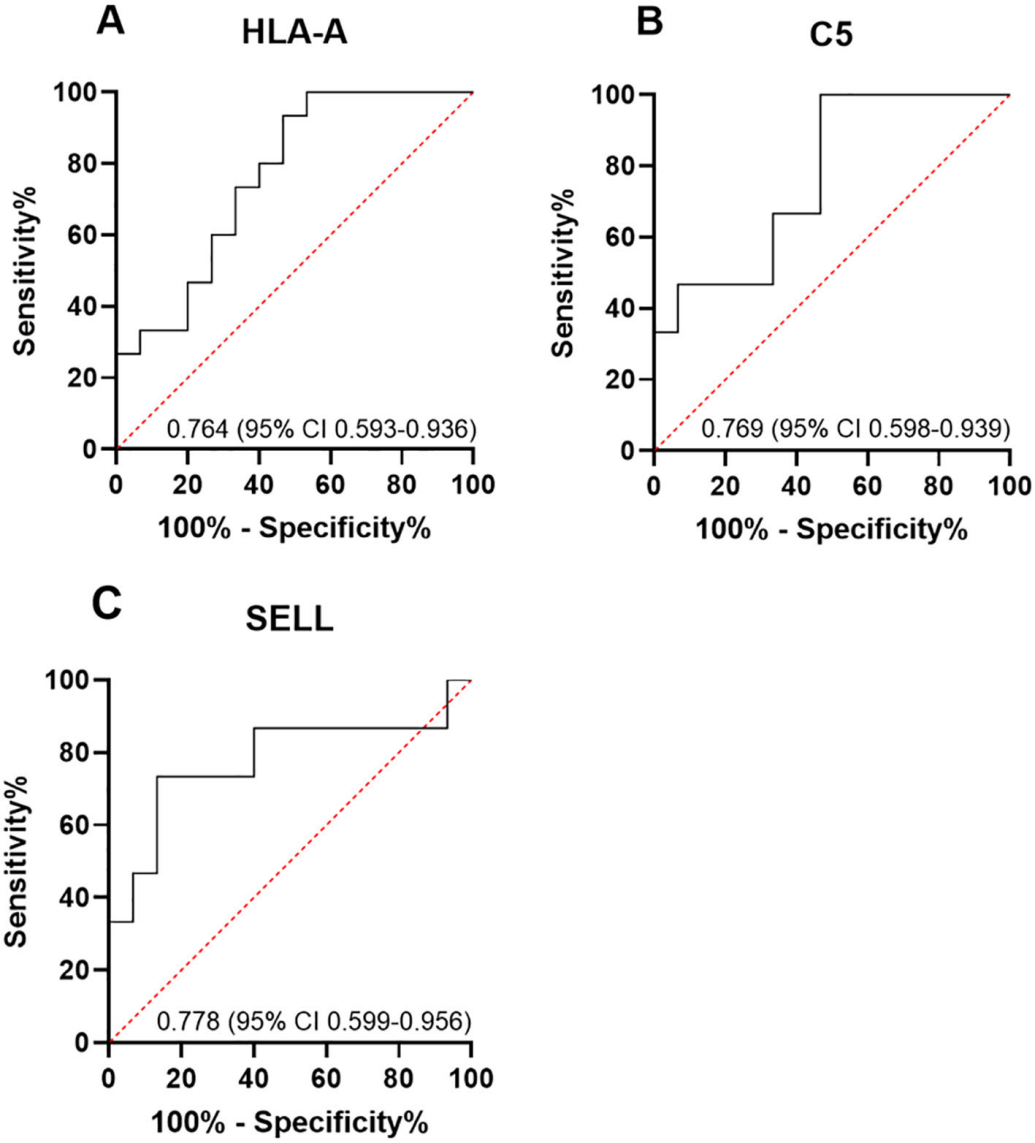
**(A–C)** ROC curve analysis of DEPs. The area under the curve (AUC) value was obtained by constructing the receiver operating characteristic (ROC) curve of differentially expressed proteins (DEPs). The AUC values of HLA-A, C5, and SELL were 0.764 (95% CI 0.593–0.936), 0.769 (95% CI 0.598–0.939), and 0.778 (95% CI 0.599–0.956), respectively.

Among the 10 downregulated DEPs, SELL has the smallest *p*-value. Among the 10 upregulated DEPs, HLA-A and C5 have relatively significant *p*-values. Combining the results of the PPI network and GO/KEGG enrichment analysis above and reviewing the literature on the association between DEPs and myasthenia gravis, the key proteins that may be related to the prognosis of thymoma with myasthenia gravis were finally identified: SELL was downregulated, and C5 and HLA-A were upregulated. The expression of the above three proteins in groups C, T1, and T2 is shown in **Figures 6A–C**: the expression levels of HLA-A and C5 in the T1 group were upregulated compared with those in the C and T2 groups, and there was no significant difference between the C and T2 groups, indicating that when HLA-A and C5 were upregulated, TMG patients had a better prognosis. The expression of SELL was negatively correlated with the prognosis of TMG. The expression level of SELL was upregulated in the T2 group with poor prognosis, and there was no significant difference between the T1 group and the C group. Thirty patients who met the enrolment criteria were selected and again divided into the T1 (N = 15) and T2 (N = 15) groups according to the grouping criteria, serum samples were collected 1 day before surgery, and the levels of the above key proteins were measured using ELISA. SELL was downregulated in the T1 group, while C5 and HLA-A were upregulated in the T1 group (**Figures 7A–C**). In order to study the performance of DEPs, the area under the curve (AUC) value was obtained by constructing the receiver operating characteristic (ROC) curve of DEPs. The AUC values of HLA-A, C5, and SELL were 0.764, 0.769, and 0.778, respectively (**Figures 8A–C**).

## 4 Discussion

In this research, the serum proteins from patients with TMG were detected by DIA mass spectrometry to find out the key proteins that may be related to the prognosis of myasthenia gravis after thymoma surgery. There were 20 DEPs between the T1 and T2 groups. Through screening, three proteins were found to be possibly related to the prognosis of myasthenia gravis after thymoma surgery: SELL (downregulated) and C5 and HLA-A (upregulated). All three proteins are expected to be potential biomarkers for predicting the prognosis of myasthenia gravis after thymoma surgery. For example, serum levels of SELL, C5, and HLA-A were detected in patients with TMG before surgery. According to our results, in patients with lower expression of SELL and higher expression of C5 and HLA-A, the long-term prognosis is predicted to be good, and the treatment plan with low treatment intensity can be selected after surgery. In patients with lower C5 and HLA-A expression and higher SELL expression, the long-term prognosis is poor or the probability of recurrence is relatively high, and a more powerful treatment plan, a higher frequency of follow-up, and a longer duration of immunosuppressive maintenance therapy are required.

The human leukocyte antigen (HLA), also referred to as the major histocompatibility complex (MHC), is a crucial component in immunology research. HLA is a major gene system that regulates human-specific immune responses and determines individual differences in disease susceptibility. It is categorized into class I, class II, and class III genes, encoding three types of antigen molecules, and is widely recognized as the foremost genetic determinant associated with susceptibility to MG (19). HLA molecules are expressed in somatic and immune cells and contribute significantly to the immune system’s response based on T lymphocytes (20). The HLA class I genes encompass the loci of HLA-A, B, and C, encoding HLA class I molecules and participating in endogenous antigen presentation.

HLA class I molecules bind with antigen peptides to form the peptide-MHC class I complex, which is present on the cell surface to stimulate specific CD8+ T cells. This is the key to initiating cellular immune responses and defense mechanisms, a pathway known as the MHC class I-restricted antigen presentation mechanism (21). MHC class I-restricted CD8+ T cells are crucially involved in autoimmune diseases, acting as either effector or regulatory and suppressor cells (22). The CD8+ T-cell population consists primarily of cytotoxic T cells (CTLs or Tc) and suppressive T cells (Ts). Ts cells have the ability to inhibit the activity of other T lymphocytes and inhibit the production of antibodies by B lymphocytes, thereby exerting a regulatory influence on both cellular and humoral immunity. The decline in CD8+ T-cell quantity and function during autoimmune disease onset or progression impairs immune response regulation, revealing that the pathogenesis of autoimmune disease involves a decrease in the quantity and functionality of Ts cells (23). The abnormal distribution of T lymphocyte subsets in MG patients is characterized by reduced CD8+ T-cell counts and an elevated CD4+/CD8+ ratio. It has been reported that when MG patients experienced exacerbation, there was a reduction in the proportion of CD8+ T cells and an elevation in the CD4+/CD8+ ratio, and during remission, the proportion of CD8+ T cells exhibited an increase, while the ratio of CD4+/CD8+ cells showed a decrease (24). Studies have shown that mice lacking MHC class I expression and having fewer CD8+ T cells exhibit more severe experimental autoimmune myasthenia gravis (EAMG) compared to controls. It was suggested that the lack of inhibitory CD8+ T cells led to an escalation in the severity of the disease (25). The immunomodulatory role of CD8+ Ts cells is restricted by MHC class I molecules (26). The HLA-A identified in this study belongs to MHC class I molecules, which were upregulated in the group with good prognosis, and the upregulation of MHC class I molecules can increase the efficiency of antigenic peptide presentation; this enhances the function of CD8+ T cells in suppressing both humoral and cellular immunity, so HLA-A may be a potential biomarker for the good prognosis of MG patients.

Research has indicated that in early-onset myasthenia gravis (EOMG), MHC class I alleles are a genetic factor within the HLA region associated with the development of EOMG (27). On these grounds, the association between MG and HLA-A in our study could be partially explained by the fact that our myasthenic patients had a mean age of less than 50 years. However, research has provided statistical evidence that MHC class I alleles correlate better with the various forms of thymic pathology, suggesting that thymic pathology is more influenced by MHC class I than by class II, with HLA-A alleles being more frequently associated with TMG, at least in the Caucasian population (28). Moreover, studies have indicated that good long-term responses in patients with MG after thymectomy are associated with HLA class I alleles (29), which is consistent with the results of this study.

Like the HLA system, the complement system also plays a crucial part in the pathogenesis of MG. Complement damages the postsynaptic membrane mainly through the classical pathway, affecting the binding of ACh to AChR on the postsynaptic membrane (30). Three activation pathways of complement converge at C3 and C5, the cleavage of C5 results in the formation of C5a and C5b, and then C5b in conjunction with C6, C7, C8, and multiple copies of C9 assembles the membrane attack complex (MAC) (31). It was found that the change in serum complement C3 level in patients with AChRab-positive MG was negatively correlated with the severity of the disease. After treatment with glucocorticoid or intravenous immunoglobulin, the disease improved, the mechanism of complement damage was reduced, and serum C3 levels gradually increased (32), suggesting that complement monitoring is helpful in determining changes in disease and prognosis. Our results showed that serum levels of C5 and C8 were upregulated in patients with TMG who had a good prognosis. From the perspective of complement consumption theory, during the pathogenesis of myasthenia gravis, a large amount of complement is involved in the immune response, leading to significant consumption of complement and thus a reduction in complement levels in the serum. In the poor prognosis group, due to more activation of the complement pathway, more C5 was cleaved and involved in MAC formation, so the levels of C5 and C8 were downregulated. In contrast, the good prognosis group showed less active complement pathways, and levels of C5 and C8 were upregulated.

The above discussion focused on the two upregulated DEPs. As the only downregulated DEP, SELL has been confirmed to have a significant association with autoimmune diseases. SELL (L-selectin, CD62L) is a single-chain transmembrane receptor glycoprotein expressed on the surface of most leukocytes, which mainly mediates selective recognition and adhesion between leukocytes and endothelial cells and plays a crucial part in lymphocyte homing and leukocyte inflammatory exudation (33). SELL is elevated in numerous autoimmune disorders, and it is significantly higher than normal in patients with systemic lupus erythematosus (34). SELL was significantly negatively associated with the disease activity and severity of skin lesions in systemic sclerosis (35). Studies have shown that CD62L+ cells can be used to reveal the dynamics of disease activity and assess T cell-mediated autoimmune and immunodeficient children’s response to drug interventions; CD62L+ T cells were inversely associated with the severity of transcription factor P3 (Forkhead box P3, Foxp3) deficiency autoimmune disease (36). Studies have found that selectins produced by activated platelets, granulocytes, and endothelial cells induce syk-dependent calcium increase, inhibit TGF-β, and lead to a decrease in Foxp3 expression; the continuous and stable expression of Foxp3 plays a crucial role in maintaining the normal differentiation and function of Treg, therefore specifically blocking the immunosuppressive function of regulatory T cells (Treg) (37). Several studies have confirmed through *in vitro* functional analysis that the function of Treg is impaired in MG, although there is no significant difference in the percentage of Treg between MG patients and healthy controls; the percentage of Treg in MG patients with poor symptom control is lower than that in MG patients with stable symptoms (38–42). This suggests that Treg may be related to the disease stability of MG (43); the percentage of Treg cells in MG patients with stable symptom control is higher than that in those with poor symptom control. The proteomic analysis results of docetaxel treatment for TMG indicated that selectin P (SELP), which is also a member of the selectin family, showed downregulation of SELP after the improvement of myasthenic symptoms after docetaxel treatment for TMG (44). In the future, it may be possible to upregulate or downregulate the expression of SELL in animal models to verify its function in patients with TMG.

Our results demonstrated that SELL is downregulated in TMG patients with good prognosis, and the prognosis of MG after thymoma surgery exhibits a negative correlation with the expression of SELL. Taking into consideration the abovementioned information, one can deduce that our findings closely align with those of prior studies on SELL in autoimmune conditions.

On the grounds that C5 inhibitors have already been included in the treatment routine of certain clinical and serological types of myasthenia gravis and that SELL and HLA-A may also become future potential anti-myasthenic targets, we trace the following directions for future studies: 1) to verify the function of key proteins in animal models, 2) to observe the impact on TMG prognosis after upregulating or downregulating the expression of key proteins, and 3) to explore the mechanisms of key proteins influencing the prognosis of TMG. The clinical translation of these biomarkers for prognosis, monitoring, and treatment will hopefully improve the outcomes of TMG patients.

While this study offers insights into the importance of SELL, C5, and HLA-A in the prognosis of MG after thymoma surgery, several limitations should be acknowledged. The group sizes are comparatively limited due to the rarity of TMG, which may potentially compromise statistical power. Large-scale, prospective studies conducted in multicenters would be preferable. The expression of proteins in different pathological types of thymoma may confound protein expression. We further conducted a stratified analysis of key protein expression in different types of thymoma. There were also differences in the expression of HLA-A, C5, and SELL (*p* < 0.05), consistent with the results of the total sample, but due to the small sample size after stratification, more samples of thymoma with different pathological types will be needed in the future for further verification. The mechanisms of how key proteins affect the prognosis of TMG need to be further explored, and clinical prediction models need to be constructed. Furthermore, an additional cohort of MG patients without thymoma could provide valuable insights into the protein expression patterns.

The moderate AUC values (0.76–0.78) for HLA-A, C5, and SELL suggested limited standalone predictive power. Combining them with other biomarkers could improve accuracy, given the previous exploration of the possible mechanisms by which HLA-A, C5, and SELL may affect the prognosis of TMG. The expression of HLA-A can be combined with the proportion of CD8+ T cells or the CD4+/CD8+ ratio, C5 can be combined with the expression level of C3, and SELL can be combined with the level of Treg. A combined evaluation may more accurately reflect the prognosis of TMG.

## 5 Conclusion

Our research not only revealed the serum protein profiles of TMG patients with different postoperative prognoses but also suggested that the expression of some key proteins may be correlated with the prognosis of TMG. The downregulation of SELL and the upregulation of C5 and HLA-A may indicate a good prognosis of postoperative myasthenia gravis. This study can provide a more accurate postoperative prognostic risk assessment of TMG patients, assist clinicians in making individualized treatment decisions, and help improve the prognosis of TMG patients. This study has a relatively small sample size. In the future, larger cohort studies are needed, as well as further exploration of the potential of key proteins as therapeutic targets. This study lays a foundation for further research.

## Data Availability

The datasets presented in this study can be found in online repositories. The names of the repository/repositories and accession number(s) can be found below: http://www.proteomexchange.org/, PXD059952.
