# Correction: Identification of potential prognostic biomarkers of thymoma with myasthenia gravis based on serum proteomics

**DOI:** 10.3389/fimmu.2025.1650718

**Published:** 2025-07-09

**Authors:** Xiaoting Lin, Peng Liu, Guoyan Qi

**Affiliations:** ^1^ Department of Oncology, Hebei Medical University, Shijiazhuang, Hebei, China; ^2^ Treatment Center of Myasthenia Gravis, People’s Hospital of Shijiazhuang, Shijiazhuang, Hebei, China; ^3^ Hebei Provincial Key Laboratory of Myasthenia Gravis, Shijiazhuang, Hebei, China; ^4^ Hebei Provincial Clinical Research Center for Myasthenia Gravis, Shijiazhuang, Hebei, China

**Keywords:** thymoma, myasthenia gravis, proteomics, prognostic, biomarkers

In the published article, there was an error regarding the affiliations for Guoyan Qi. As well as having affiliations 1,3,4, she should also have “2 Treatment Center of Myasthenia Gravis, People’s Hospital of Shijiazhuang, Shijiazhuang, Hebei, China”. Also, the order of authors in the author list of the published paper was erroneously given as:

“Xiaoting Lin, Guoyan Qi, Peng Liu”

The correct author list reads:

“Xiaoting Lin, Peng Liu, Guoyan Qi”

The original article has been updated.

